# Gay and bisexual men’s views on reforming blood donation policy in Canada: a qualitative study

**DOI:** 10.1186/s12889-019-7123-4

**Published:** 2019-06-17

**Authors:** Daniel Grace, Mark Gaspar, David Lessard, Benjamin Klassen, David J. Brennan, Barry D. Adam, Jody Jollimore, Nathan J. Lachowsky, Trevor A. Hart

**Affiliations:** 1 Dalla Lana School of Public Health, University of Toronto, 155 College Street, 5th Floor, Room 556, Toronto, ON M5T 3M7 Canada; 2 Dalla Lana School of Public Health, University of Toronto, 155 College Street, 5th Floor, Room 510, Toronto, ON M5T 3M7 Canada; 3 Centre for Health Outcomes Research, McGill University Health Centre, 5252 de Maisonneuve West, Montréal, QC H4A 3S5 Canada; 4 Department of History, Simon Fraser University, 8888 University Drive, Burnaby, BC V5A 1S6 Canada; 5 Factor-Inwentash Faculty of Social Work, University of Toronto, 246 Bloor St W, Toronto, ON M5S 1V4 Canada; 6 Ontario HIV Treatment Network, 1300 Yonge Street #600, Toronto, ON M4T 1X3 Canada; 7 Community-Based Research Centre, 1007-808 Nelson Street, Vancouver, BC V6Z 2H2 Canada; 8 School of Public Health & Social Policy, Faculty of Human & Social Development, University of Victoria, Michael Smith Foundation for Health Research Scholar, P.O. Box 1700, STN CSC, Victoria, BC V8W 2Y2 Canada; 9 HIV Prevention Lab, Department of Psychology, Ryerson University, 350 Victoria St, Toronto, ON M5B 2K3 Canada

**Keywords:** Men who have sex with men, Blood donation policy, HIV, Qualitative, Canada

## Abstract

**Background:**

Researchers and activists have long called for changes to blood donation policies to end what is frequently framed as unjustified bans or deferral periods for men who have sex with men (MSM). Since 2016, in Canada, a man had to be abstinent from all sexual contact (anal or oral sex) with other men for at least 12 months in order to be an eligible blood donor. As of June 3, 2019, this deferral period was reduced to 3 months.

**Methods:**

To better understand the acceptance of existing deferral policies and possible future policy, we conducted 47 in-depth interviews with a demographically diverse sample of gay, bisexual, queer, and other men who have sex with men (GBM) in Canada’s three largest cities: Vancouver, (*n* = 17), Toronto (*n* = 15), and Montreal (*n* = 15). Interviews were coded in NVivo 11 following an inductive thematic analysis. We focus on men’s preferred policy directions and their opinions about a policy change proposed by Canada’s blood operators: *a 3-month deferral for all sexual activity between men.* We interviewed GBM approximately one-year before this new deferral policy was approved by Health Canada.

**Results:**

Most participants were opposed to any deferral period in relation to MSM-specific sexual activity. A fair and safe policy was one that was the “same for everyone” and included screening for several risk factors during the blood donation process with no categorical exclusion of all sexually active MSM. Participants believed that multiple “gender blind” and HIV testing-related strategies could be integrated into the blood donation process. These preferences for a move away from MSM-specific exclusions aligned with their opinions concerning the possible change to a 3-month MSM deferral, for which participants shared three overarching perspectives: (1) *step in the right direction*; (2) *ambivalence and uncertainty*; and (3) *not an improvement.*

**Conclusion:**

A predominant assertion was that a change from a 12-month to a 3-month deferral period would not resolve the fundamental issues of fairness and equity affecting blood screening practices for GBM in Canada. Many participants believed that blood donation policy should be based on more up-to-date scientific evidence concerning risk factor assessment and HIV testing.

**Electronic supplementary material:**

The online version of this article (10.1186/s12889-019-7123-4) contains supplementary material, which is available to authorized users.

## Background

Multiple physiological and behavioural factors can disqualify a person indefinitely from donating blood in Canada or lead to a temporary donation deferral—a period of time during which one is not eligible to donate blood. The current criteria for blood donation deferral range from being relatively uncontroversial, such as having had a dental cleaning in the last 24 hours or having had a tattoo or piercing in the last 3 months, to being highly contested, such as deferral practices for men who have sex with men (MSM) [1]. Since 2016, in Canada, a man had to meet all other eligibility criteria in addition to the following: “their last sexual contact with another man (anal or oral sex) was 12 or more months ago” [2]. As of June 3, 2019, this deferral period was reduced to 3 months [3].

Despite their ubiquity internationally, these MSM-specific deferral practices remain greatly disputed [4–9]. For over 20 years, Canadian Blood Services (CBS) along with Héma-Québec, its sister organization operating in the province of Québec, have been Canada’s blood operators, a role they took over from the Canadian Red Cross Society in 1998. Blood donation “lifetime deferrals” or “indefinite deferrals” for MSM were first introduced in Canada as a response to the AIDS epidemic and the tainted-blood scandal [10–12]. It is estimated that approximately 2000 people in Canada, many of whom were hemophiliacs, contracted HIV through the blood supply, and many more with hepatitis, resulting in a billion-dollar compensation package for those affected [11, 13]. Orsini and colleagues [14] elucidate the legacy of this tragedy:

When news spread that Canada’s blood system was compromised and thousands acquired HIV, and later hepatitis C, as a result of government wrongdoing, the idea that HIV had affected so-called “innocent” blood transfusion recipients gave way to “othering” dynamics that marked them off from groups perceived as morally culpable, including gay men, people who use drugs, and sex workers (see [15], p9)*.*

This initial policy response of an indefinite deferral for some groups including MSM was remarkably durable in Canada, remaining unchanged until 2013 when the donation policy was altered to a requirement of 5 years of abstinence from any sexual activity between men [16]. In 2016, based on research demonstrating the safety of a 12-month deferral [17], this period was reduced again—indicative of trends across many counties including the United States—to a 12-month deferral for men who have sex with men [18]. The time between HIV infection and the ability for testing technology to confidently detect the virus in collected blood were long considered by CBS to be significant limiting factors in their ability reduce the deferral period [2]. CBS has since cited more recent advancements in HIV testing as rationales motivating their reduction in deferral periods [2]. CBS has framed the 12-month deferral period as a “waiting period” serving as an “incremental step” in updating its donation criteria [2]. Echoing this language, when describing the 2019 policy change to the reduced timeframe of a 3-month deferral, CBS chief executive officer explained that “this further reduction to the waiting period represents the next available step forward in updating our blood donation criteria” [3].

This policy reform to a 3-month period of sexual abstention has occurred within a shifting landscape of biomedical HIV prevention [19] and knowledge of the epidemiology of sexually transmitted and blood infections (STBBIs) in Canada. GBM continue to experience a disproportionate burden of STBBIs including new HIV infections in Canada, accounting for over half of Canada’s ~ 2000–3000 new HIV infections each year [20]. GBM’s relative risk of contracting HIV is 131 times higher than that of other men in Canada [21] and represent nearly half of all HIV cases in the country (49.1%) [20].

Beyond epidemiological considerations, blood donation policy has sparked debates regarding citizenship and the social significance of donation. In his classic work *The Gift Relationship,* which traces social, economic and political forces in relation to blood donation internationally, Titmuss [22, 23] describes the deep symbolism and significance attached to blood historically. Blood donation has been a mark of responsible citizenship and altruism—to not be able to donate is, by consequence, a way of restricting citizen involvement [24]. Contemporaneously, blood donation is frequently framed using a “gift of life” discourse [25]. CBS media campaigns have previously articulated: “*it’s in you to give*”—an apparent moral obligation and appeal to the altruistic Canadian donor. Recent promotions of blood donation in Canada have also positioned the giving of blood as part of a collective social action.

Researchers and activists have called for further policy changes to rethink donation deferrals specific to MSM, frequently citing these blanket deferrals as being homophobic, discriminatory, and illogical [8, 26, 27]. There has been a significant debate in Canada and internationally for decades on what blood donation policy should look like [12, 26, 28, 29]. Some have argued that these deferral practices are in fact ineffective at blocking at least some MSM from donating [30]. Collecting the best available epidemiological and biomedical evidence to ensure a safe blood supply is a clearly stated priority of CBS and Héma-Québec. CBS provides an overview of how blood safety is a primary priority at “every step of the process” from donor screening and testing to production and storage [31]. Canada’s blood operators have also stated the importance of the opinions and trust of communities affected by current and future policies—both donors and recipients—to understand how the policy will be understood and accepted. If people reject the legitimacy of a policy, this can impact the degree of trust they put into public institutions [32].

The primary objectives of our research were to understand GBM’s attitudes toward and acceptability of the blood deferral policy in place at the time of the interviews (12-month deferral), as well as their opinions on possible reforms to this policy. This work was funded as part of a strategic initiative aimed at generating evidence to inform alternative blood and plasma screening deferral practices for MSM while maintaining the safety of the blood supply. To meet these objectives, we conducted in-depth qualitative interviews with a demographically diverse sample of gay, bisexual, queer and other men who have sex with men (GBM) in Canada’s three largest cities: Vancouver, Toronto, and Montreal. We understand this group to be heterogeneous with unique perceptions regarding existing and future blood donation policies. In this paper, we elucidate these men’s preferred policy directions for MSM as well as their opinions about a 3-month deferral for all sexual activity between men [2].^1^

Qualitative research has been conducted with GBM living in the United Kingdom regarding the life-ban deferral and 5-year deferral policy and has explained that these men viewed these policies as “inequitable, discriminatory, and, above all, lacking a clear rationale” [33]. While a 3-month blood donation deferral policy has been recently introduced in other countries, including the United Kingdom [28], no qualitative research has been published in Canada on GBM’s perspectives on the 12-month policy or the newly introduced 3-month policy change that shortens the deferral window but maintains a specific focus on MSM.

## Methods

### Participant selection

We conducted 47 in-depth qualitative interviews [34] with GBM living in Vancouver (*n* = 17), Toronto (*n* = 15), and Montreal (*n* = 15) who were recruited from the Engage study. Engage is a large sociobehavioural and biomedical study focusing on GBM health, HIV, and sexually transmitted and blood borne infections (STBBIs). Potential qualitative study participants who completed the quantitative components of Engage and agreed to be contacted for additional studies were emailed and asked if they would be interested in being interviewed for this qualitative study.

Three dimensions were considered while recruiting participants (Table 1). First, we focused on capturing diversity in terms of age and ethno-racial background. Second, we recruited a heterogeneous sample of HIV-negative participants in terms of their sexual risk profile, which were determined by using participants’ responses to the Engage quantitative questionnaire in order to calculate their HIV Incidence Risk Index for Men who have Sex with Men (HIRI) scores. HIRI is a metric determining a participant’s relative risk for contracting HIV based on his reported age as well as his sexual and substance use behaviours [35]. Our interest in using HIRI scores was to ensure that we were speaking with men with varied sexual behavioural profiles. Lower sexual risk profiles included men with HIRI scores of less than 10, medium risk were men with scores between 10 and 15, and higher risk participants had HIRI scores of greater than 15. We paid particular attention to gathering the perspectives of men in the “lower risk” category who might be more likely to become eligible for donating blood under modified policies. Third, we recruited several HIV-positive men in each city to capture a comprehensive view of how blood donation policy affects GBM communities, including its connections with HIV stigma. Research ethics approval was provided by the research ethics boards of the University of Toronto, Ryerson University, the University of Windsor, McGill University, the University of British Columbia, Simon Fraser University, and the University of Victoria.

**Table 1:**
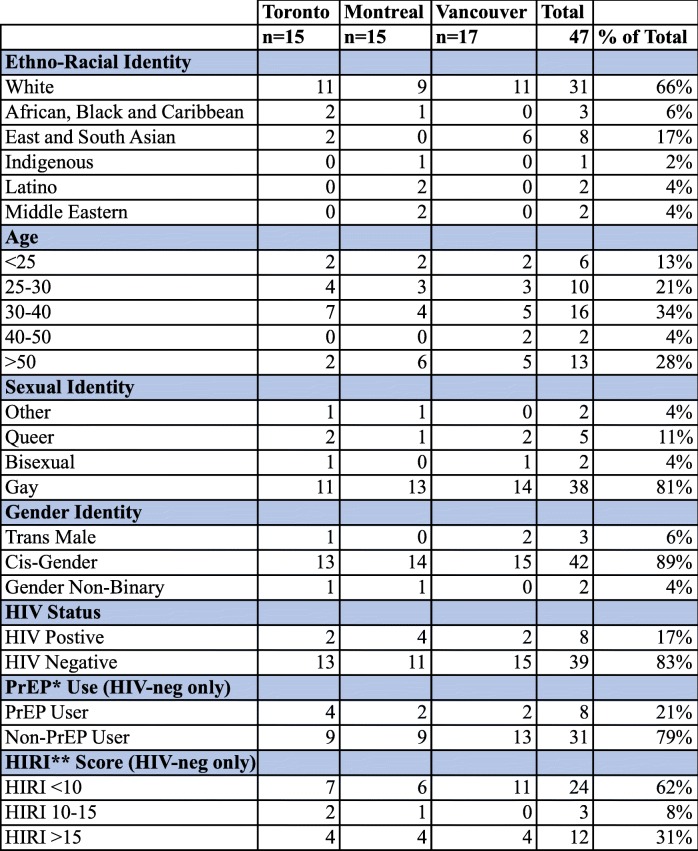
Sociodemographic and Behavioral Characteristics of Study Participants (*n* = 47)

* Pre-Exposure Prophylaxis (PrEP); **HIV Incidence Risk Index for MSM (HIRI-MSM)

### Data collection

The one-on-one interviews were conducted at university campuses, study offices, or community-based organizations in the three cities. An interview guide was developed in consultation with the research team and the three community engagement committees operating in Vancouver, Toronto, and Montreal (Additional file 1). The community engagement committees were made up of service providers and members from local GBM communities who were responsible for advising on Engage’s study design and implementation, as well as offering input on analysis plans. While interviews in Toronto and Vancouver were conducted in English only, interviews in Montreal were conducted in French or English depending on the preference of the participant. In the results below, we have translated data from the French language interviews into English. The interviews followed the interview guide closely to ensure consistency across the three cities. Participants provided informed consent before the interviews. The interviews lasted between 30 to 90 minutes and were digitally audio recorded.

The interview guide had six overarching domains: 1) *introductions, socio-demographics, and rapport building*; 2) *policy comprehension and general opinions on blood donation*; 3) *past experiences with blood donation*; 4) *opinion on potential policy changes (*e.g.*, gender-blind screening, 6-month deferral, and 3-month deferral)*; 5) *participant’s evaluation of personal risk levels for contracting HIV and STIs and interest in donating blood under modified policies*; and 6) *opinions about existing and modified screening questions and deferral procedures*.

The interviewers created and shared detailed post-interview notes outlining key reflections from each interview. The interviewers and first author met during the data collection process to discuss these notes, ensure that recruitment objectives were being addressed, and to consider emerging themes, including any ideas that were not explicit in the interview guide.

### Analysis

The interviews were transcribed verbatim and reviewed for accuracy. Transcripts were entered into QSR NVivo 11 software and coded using thematic analysis [36]. This process of coding consisted of three main steps. Step one involved becoming acquainted with the interviews. This was achieved by conducting interviews and reading interview notes and transcripts. Step two involved generating initial codes and broad categories as a team to begin organizing the material into more digestible sections. Step three involved defining, naming, and explaining themes (recurrent patterns and meanings in the data). Preliminary analysis and results were shared with the research team, with special attention to monitoring trends across the three cities to ensure that important context-specific nuances were not missed. As part of our integrated-knowledge translation strategy, preliminary results have also been shared with CBS, community partners including the Community-Based Research Centre (CBRC) in Vancouver, and at scientific conferences.

While all aspects of the interview were considered in the analysis process, the following results focus closely on two main themes: 1) *participants’ general reactions to blood donation policy for men who have sex with men, including their perspectives on the relationship between equity, policy, and science*; and 2) *participants’ reactions toward specific alternative models of blood donation, including gender-blind screening and a 3-month deferral period*.

## Results

### Awareness of blood policy history

Participants’ awareness of the history of MSM-specific blood donation policies in Canada varied. The majority of men explained that they knew that there used to be a lifetime ban on blood donation for MSM. However, only a few participants in each province mentioned being aware that there were cases where HIV was transmitted through blood transfusions early in the epidemic. Perhaps not surprisingly, many of these participants tended to be older, indicating a generational component to the perspectives and knowledge on policy history among some men who were adults that lived through the early years of the AIDS epidemic.

Most participants believed that the original lifetime ban was a result of insufficient testing technology, homophobia, and AIDS stigma. For example, one participant stated that the reason for the indefinite ban had “something to do with maybe religion or just the stigma on homosexuality” (age 26, HIV-negative, Toronto), while another claimed, “I think that at that point it was mostly fear and paranoia that caused [the life ban] but since then there’s been a lot more testing and research done to disprove it [as necessary]” (age 34, HIV-negative, Toronto). One interviewee made it clear how discriminatory the original life ban was toward GBM communities: “It just seemed really stigmatizing and growing up and hearing in the community, ‘It’s in you to give and donate. It’s a good thing to do. It helps people and we need blood.’ And then to be completely shut out based on your sexual identity is really stigmatizing and really disappointing” (age 39, HIV-negative, Vancouver).

Despite differences in awareness of the history of MSM blood donation policy, all of the participants knew of the 12-month deferral policy, which was in place at the time of the interviews. They were, in general, quite eager to express their views on whether they considered this policy equitable and what policy alternatives they thought to be improvements to the current practice of deferment. Below, we outline participants’ views on these questions, beginning with their perceptions of the 12-month deferral policy.

### Policy equity, scientific evidence, and policy improvements

Most participants expressed that they did not consider the 12-month abstention-based policy for MSM to be equitable. They believed that a fair and safe policy would be one that was the same for all people. For example, one man communicated that the ideal policy would be one where “men who have sex with men [are] allowed to donate blood freely” (age 24, HIV-negative, Toronto).

Viewpoints about policy equity were guided by a general belief that differences in policy rooted in sexuality or identity, or behaviours closely aligned with identity, are inherently unjust and discriminatory. As one participant stated, he wanted “a policy that is equitable and the same for everyone regardless of their sexual orientation, their gender identity, their colour, or their origins and cultural background. I think that the policies now in place across the board are very prejudiced” (age 33, HIV-negative, Vancouver). Another man declared that “If there is a period of exclusion for a gay man, there must be the same thing for other categories of people. And also, if there is not for other categories of people, there must not be [one] for gays” (age 43, HIV-negative, Montreal). Echoing a desire for equitable policy, one participant put it this way: “Yeah, I just think it should be equal. It should be equitable. It should be the same language as what it is for straight people. [ …] just like HIV would affect a straight person’s body the same way it would a gay person’s body” (age 33, HIV-negative, Toronto).

A few participants also commented that having low risk sex like oral sex as an exclusion criterion did not make sense to them: “Well my understanding is that they can now donate blood if they’ve been celibate for a year. What I’m confused about is what they mean by “celibate” because there’s a whole range of sexual practices. Some of them are extremely low risk” (age 33, HIV-negative, Vancouver). Another stated: “if somebody gave somebody head [i.e. oral sex] three months ago, I still think they should be able to donate” (age 27, HIV-negative, Toronto).

A minority of participants thought that a MSM-specific blood donation policy was not problematic. For example, one man described MSM-specific blood donation policies as necessary for public safety given higher rates of HIV among gay men:

Well I think it’s statistically proven that gay men do have higher rates of HIV infection compared to straight people, and also they are statistically having more sex and more dangerous sex than other populations. And I think that as a public safety concern we should place some kind of screening to protect the public. And if the period of HIV infection being undetectable didn’t exist, I wouldn’t have these concerns. But considering that gay people are at a higher risk and there is a period where it’s undetectable, I feel uncomfortable without that kind of [timed abstention] rule [for MSM] (age 26, HIV-negative, Toronto).

Nonetheless, a large majority of participants believed that any MSM-specific policies were discriminatory and that a more equitable policy choice would be one that better matched with current scientific evidence. For example, one interviewee declared that while MSM-based deferral policies made sense previously, they are now outdated: “It’s completely unfair, there is no reason today. There is no reason anymore” (age 43, HIV-negative, Montreal). Another articulated that the policy does not reflect advancements in technology: “The technology is changing. The science is changing. The screening methods are getting better and better and better, so I think our policy needs to reflect that” (age 39, HIV-negative, Vancouver).

These men did not believe that MSM-specific policies were based on the best available science and argued that current deferral policies could be improved to increase the donor supply in a more equitable fashion if they were more closely aligned with scientific evidence and technological advancements. Many articulated how an MSM-specific policy appeared to be based on outdated knowledge and logic that they did not agree with or understand. Thus, opinions about equitable policy and scientifically informed policy were mutually intertwined. For example, one man stated:

I think strong intake and then blood testing that has absolutely no distinguishing between gay men, men who have sex with men and heterosexual, bisexual, pansexual people because it’s based on historical fallacies. Again, nothing has ever been presented to me that has convinced me that the ban against men who have sex with men’s blood makes any sense at all (age 30, HIV-negative, Toronto).

Participants discussed two general ways in which the policy could catch up to existing scientific knowledge on HIV and, as such, could also become more equitable. The first was through universal or “gender-blind”^2^ screening practices focusing on risk factors for all blood donors regardless of their sexual orientation. The second was through deferral practices closely guided by HIV/STI testing technologies.

#### Universal screening and deferral practices: gender-blind screening

Although most participants agreed that MSM are more likely to acquire HIV, with some also referencing increased hepatitis and syphilis transmission, many expressed opinions about deferral-based policies that suggested that these policies were outdated and misinformed about sexual health realities. These opinions were closely connected to reflections on how screening and deferral practices should instead take into consideration a more sophisticated measure of transmission factors that could be equally applied to any sexually active person (regardless of sexual orientation or the gender of their partner). These participants argued that the screening and donation processes should include asking more detailed questions and offering counselling about transmission factors, sexual behaviour, preferred prevention strategies, and recent testing history.

For example, one interviewee declared that the ideal policy would pay more attention to “risk behaviour as opposed to sexuality” (age 34, HIV-negative, Toronto). Echoing this theme, another stated that “we can rely on a valid assessment of the person’s risk level and the fact that they’re gay or not is irrelevant” (age 69, HIV-negative, Vancouver). Some participants prefaced their reflections on what things would look like from a policy perspective in an “ideal” world. For example, one man put it like this:

In an ideal world, then, the rules should be the same for everybody, because straight people get HIV, they get STIs, they’re not always monogamous, they have oral sex, they have anal sex. […] So, it should be the same. I would say make the rules the same for everybody (age 61, HIV-negative, Vancouver).

Some participants articulated that they simply did not understand why blood donation policy was different in instances of opposite-sex versus same-sex sexual activity. For example, one man said: “I feel like it should be exactly the same as straight couples. Whatever rules are in place over there should be fine for us because it’s ‘why’ at this point?” (age 24, HIV-negative, Toronto). A recurrent theme among some participants was the feeling that it is unfair and/or unclear why heterosexuals can have frequent casual “unsafe” sex and still donate blood while they themselves are not able to donate blood when practicing safer sex strategies and/or being in monogamous relationships. One participant added:

We should look more at risky sexual behaviour independently of a person’s sexual orientation or gender. […] Get rid, in fact, we have to extract that thing about sexual orientation in this policy. We are talking about blood, not about sexual orientation. You know, my blood is not supposed to be better or worse. (age 34, HIV-negative, Montreal)

While these participants thought that everyone should be screened based on the same set of “risk factors,” they were generally vague or uncertain on what exactly these risk factors should be. Moreover, some participants made it clear that members of GBM communities have higher probabilities of contracting HIV, contradicting the idea that condomless sex between two heterosexuals versus two men is identical in terms of risk and opposing the notion that risk behaviours can be evaluated independently of the gender of the donor and their sexual partners. Moreover, participants did not speak to how applying a universal risk factor policy which excluded, for example, all individuals based on recently having had condomless sex within a specific time window, could drastically reduce the current heterosexual donor pool.

#### Relying on HIV/STI testing

Participants discussed how they saw MSM-specific donation policies as inequitable since they did not seem to consider scientific advancements in HIV testing. As one man expressed, a 12-month deferral did not make sense to him because current testing technologies are accurate by 3-months:

Well, we have to evolve with science. If we can, you know, with the efficiency of tests at this moment, I don’t know, we say 3 months, but I read things, it was like 2-3 weeks, they can detect, I don’t know. So policy should evolve with science, if we can detect and minimize risks for others, I would agree with that. Anyway, for me, it’s like this: follow science. (age 35, HIV-negative, Montreal)

The men we interviewed demonstrated varying levels of knowledge regarding how blood donation works in practice. Some participants were unsure whether or not CBS or Héma-Québec relied solely on a potential donor’s answers to the screening to determine the safety of collected blood. Similarly, others expressed confusion about the current 12-month deferral policy because they did not understand why CBS or Héma-Québec had to defer donations based on screening questions if they were going to test all of the blood regardless. Several participants argued that CBS and Héma-Québec should accept all blood donations and then test the blood prior to using it for transfusions, since relying on people’s answers to screening questions is not adequate.

As one interviewee stated: “I’m presuming that at some point they’re testing the blood so if your blood is fine then I don’t see why it’s relevant who you’re having sex with” (age 23, HIV-negative, Vancouver). When asked about screening questions, one man mentioned, “I think it’s irrelevant [to ask screening questions]. It is not the nurse’s business. Once again, the test is the best thing” (age 22, HIV-negative, Montreal). While another declared that “I think they [sexually active persons] should both donate blood and it should just be tested” (age 26, HIV-negative, Toronto).

Several participants articulated that a point-of-care HIV test or a mandatory HIV/STI test should be a part of the blood donation process. For example, one participant described how he thought this could work in practice:

I think that it should be rapid point of testing at the donation site. If you test [HIV] positive, you’re ineligible. They refer you to see a doctor or tell you to go see your family doctor or schedule an appointment for you to go see your family doctor. Whatever it is that they do, I feel like they should ask you questions in regards to your sexual history, especially for like intravenous drug use and your general kind of health. But at the end of the day the sex that you have shouldn’t really play a part if you’re eligible to give blood (age 34, HIV-negative, Toronto).

Interestingly, while the above participant was critical of sexual behaviour being a factor in determining donor eligibility, he did not consider the potential for safer drug use and agreed with a blanket deferral for intravenous drug users. Most participants we interviewed thought that the blood donation process could become more equitable by asking questions about past HIV testing history, accepting all blood donations and testing the blood prior to use, and/or having a mandatory point-of-care HIV/STI tests as part of the blood donation process.

### Considerations of a 3-month deferral policy

We asked participants about their opinions on a donation policy shift from a 12-month to a 3-month abstention for sexual activity between MSM *before* this 3-month policy was implemented. Viewpoints about this proposed 3-month deferral policy reflected the participants’ general opinions about deferral policies specific to MSM, as discussed above. The majority who believed that any MSM-specific policy was discriminatory and inequitable continued to consider a 3-month deferral to be an inadequate policy change. However, though most did not consider a 3-month deferral to be an ideal policy, there were variances in how participants understood the potential benefits and limitations of this proposed change. That is, while the opinions discussed above represented more general views on the relationship between equity, science, and policy-making—and thus offered somewhat open-ended and general reflections on policy improvements—the 3-month option was a clear and specific policy alternative that caused participants to evaluate issues of equity and scientific evidence in relation to pragmatic aspects of policy-making, including the notions of compromise and incremental improvement.

Participants expressed three general viewpoints about a 3-month deferral policy: *Step in the Right Direction*, *Ambivalent or Uncertain about Implications*, and *3-Month Deferral not an Improvement.* The last viewpoint also included the sub-category *3-months not being long enough of a deferral.* A connecting thread across these policy perspectives was that this policy change would not be able to resolve the fundamental issue of inequity currently affecting MSM blood screening practices in Canada.

#### Step in the right direction

Several participants vocalized that they considered a 3-month deferral to be a positive (albeit imperfect) policy change. Though they did not consider this to be the ideal policy, they understood this to be an incremental step in the right direction. For instance, one man reacted to the proposed policy change with “Wow. Getting half shorter [than 6 months]. That will be even more positive” (age 67, HIV-negative, Vancouver). He mentioned that this would make more people eligible to donate. A few interviewees described the 3-month deferral as being “okay” or “a good idea” as this period of time would more closely approximate the window period of current HIV testing technologies.

One participant described the 3-month deferral as a pragmatic “stepping stone”:

I think [a 3-month deferral is] a little bit more realistic. There probably could be an even better policy but if we’re talking about [being] realistic and making progressions that would be a big step compared to the one that’s in place right now and I think it’s a lot more realistic for more individuals if they think that donating blood is a high priority for them. (age 22, HIV-negative, Vancouver)

This participant went on to clarify that more research is needed to improve the policy and that “the work shouldn’t just stop there [3 months]. There should be more consideration into not having a time policy, and screening based on sexual practices…”.

One participant expressed that he saw the 3-month deferral as a real improvement on either the 12-month or a potential 6-month deferral, because there would be more people eligible to donate. He described the 3-month policy change in positive terms: “So I feel like it definitely would be better if that happened. It would just be one step closer to becoming like good for everyone” (age 26, HIV-negative, Toronto). While another man stated: “It’s more reasonable, you know. At least, it fits with something we hear often, that is, after 3 months, you are sure and certain that you did not get anything if you did expose yourself to a risk” (age 35, HIV-negative, Montreal).

#### Ambivalent or uncertain about the implications

While the previous category positioned a 3-month deferral as a productive, incremental compromise, this group was far more uncertain about whether or not this policy shift signified any real improvement. For example, one participant mentioned that “It does seem like it’s a lot of the same. I guess comparatively three months is better than a year. But putting a time limit on it versus actual life practices is kind of backwards thinking” (age 26, HIV-negative, Toronto). One man argued that this policy shift would probably increase the blood donor pool with a fairly good-sized group, but he ultimately argued that such a policy “in all actuality does not affect whether or not I’m eligible or not eligible if I’m meeting all the criteria that’s set. So why is there a time limit?” (age 30, HIV-negative, Toronto).

Some participants who expressed degrees of uncertainty with this policy change tried to determine the extent to which this reform may increase the eligible donor pool of MSM. For example, one participant believed that the policy shift would broaden the pool of eligible donors but would still fail to reach most people who are regularly sexually active. Hence, he reflected on how such a change would be “a step in the right direction but not one hundred percent” (age 34, HIV-negative, Toronto). Some participants tried to balance both sides of the argument for this policy shift, ultimately appearing ambivalent about a policy change.

One participant struggled to weigh the practicality of a 3-month abstention-based policy that aligns better with what we know about testing, with his strong desire for universal non-MSM specific based deferral practices:

Well, 3 months. That’s difficult. For me, it coincides a little with screening [i.e. HIV testing]. That is, you know, I mean, I imagine in 3 months, well, as I say, maybe 3 months makes more sense, but still, having sex with a man should not be a criterion for exclusion (age 34, HIV-negative, Montreal).

Multiple participants appeared to understand the idea of a 3-month deferral period—or window period—given how this length of time is part of HIV and sexual health testing culture.

One man first expressed mild interest for the 3-month deferral and said that he would be able and willing to donate under this policy. However, when he asked if this 3-month deferral would affect all potential donors and was told that it would remain specific to men who have sex with men, he opined: “Oh okay. No, that’s so unfair. Then three months is still not fair” (age 24, HIV-negative, Vancouver).

Another similarly stated that he did see the 3-month policy to be an “improvement” but his ambivalence was pronounced: “Yeah. I guess if there was a lack of other options, I would rally behind it” (age 30, HIV-negative, Toronto). This participant mentioned that the deferral policies did not “make any sense to me” and that he was cautious about how his own prejudices toward HIV and higher risk sex may be affecting his evaluations of this potential policy change. Similarly, one interviewee thought the 3-month policy would “be productive I think. I mean if the only possible way to approach it is to approach it from the perspective of an abstention period” (age 23, HIV-negative, Vancouver). Yet he was still unclear as to why this particular temporal change:

I’d be interested to know what the buffers are. Why is it 3 months for example? What’s the rationale there? Is it just to be on the safe side? Because I feel like I need to know more. I feel like that cannot just possibly be it. But yeah, I think three months is more likely to be successful but still it is again a long time and it’s a lot to expect of people in terms of to dictate that people would want to [donate]. (age 23, HIV-negative, Vancouver).

One participant considered the 3-month deferral to be “a little bit more realistic” than the current policy (age 34, HIV-negative, Vancouver). However, he did not understand the logic whereby some people could donate blood and be sexually active and other people had to abstain. In his opinion, “given the testing that we have today it should just be like no sexual contact for X period of time for anyone donating blood. You know, probably like a month, I tend to think.” Meanwhile another participant described the 3-month deferral as being “more like possible” (age 27, HIV-negative, Vancouver) to increase his eligibility. However, he argued that he had worked hard to overcome shame around sex and body image issues. Being sexually active was really important to him because “it’s healing, it builds community and it’s fun. So I don’t think, you know, 3 months, I’ve certainly gone that long [without sex] but I still don’t think it, like I think there’s, I guess I’m a very sex positive person and any sex negative policy irks me the wrong way, or rubs me the wrong way. It irks me.” This participant echoed a theme common across many interviews: MSM blood deferral policy is anti-gay sex.

#### Not an improvement

The last viewpoint was that the 3-month deferral policy was not a significant improvement on the current 12-month deferral policy. These participants were exceedingly critical of any policy change that further differentiated between homosexual and heterosexual sexuality. One man argued that “abstinence isn’t the solution” (age 33, HIV-negative, Vancouver) and another mentioned “I don’t think the [reduced] timeframe makes any difference” (age 69, HIV-negative, Vancouver). Many participants argued that a 3-month abstention policy is still an inequitable policy. One man put it like this:

For me, it’s discrimination. This is an injustice we must correct it. Do we say it’s 3-months for everybody, straight people as well, we want that waiting period as well after intercourse? Fine, but also for straights (age 43, HIV-negative, Montreal).

Many participants made it clear that a 3-month deferral leaves us in the same situation as a 12-month deferral: a policy that discriminates. One participant expressed his concern like this: “Because you are homosexual, you have to wait 3 months, 6 months? And the straight couple, they don’t wait? I mean, the man and the woman, the woman, she can have 4 partners in one evening, and the next day, she will give blood. But us, because it’s ok with the straights, you’re homosexual, you wait 3 months. I don’t follow” (age 65, HIV-negative, Montreal).

Some interviewees were critical of the 3-month policy because they did not see it changing anything since they were not going to go 3 months without any sexual activity. As one man described, the 3-month deferral would be “still problematic” because “who’s going to be celibate for those periods of time? Full stop” (age 36, HIV-positive, Toronto). Meanwhile, another participant questioned whether it was realistic or impactful because “you may [just] find the occasional person that will do that” (age 59, HIV-positive, Vancouver).

For one man, the 3-month deferral does nothing to address the equity issues key to the current debates on blood donation:

I have a problem with the deferral because it’s still aiming at the gay population but the bisexual and the straight man who had unprotected sex [with women] and goes to the clinic and gives blood has an easier time than us. So I have a problem with this. (age 63, HIV-positive, Toronto).

Similarly, one interviewee described the 3-month policy as “unrealistic,” mentioning that no one was going to want to donate under that policy and describing the 3-month deferral as:

A bit of a slap in the face because it’d be straight up ignorant. It’s not actually looking at the [sexual] partnership. Again, pulling up to a [gay] couple that’s been together for 20 years and being completely monogamous— for some reason they have to stop having sex for 3 months just [because] the blood might be tainted. What? Like, it’s not realistic in the slightest. (age 24, HIV-negative, Toronto)

Another participant argued that the 3-month deferral would only be a good choice if “it was applied to heterosexuals as well and if it’s the same rules for everyone then yes but if it’s just targeting gay men I’d say no” (age 49, HIV-negative, Vancouver). This participant described the policy change as somewhat impractical and not a significant change. Many men expressed that they did not think moving to a 3-month deferral would increase the blood supply by much given the improbability of most people remaining sexually abstinent.

The following participant was skeptical of whether or not service providers at blood donation clinics would actually want to take the blood donation of any MSM under a 3-month deferral policy because it would be difficult to prove that men had actually been abstaining:

I’d be interested to know what that ends up looking like in terms of service providers even in that scenario where it’s a 3-month testing abstention period, would service providers actually even want gay men’s blood. Would they believe them enough? Would it be a pool of candidates who could be trusted enough in terms of the accuracy of the information provided? That’s a question I would ask, yeah. (age 23, HIV-negative, Vancouver)

#### Three months not long enough of a deferral

Four participants expressed criticisms of the 3-month deferral because they did not think it was long enough of an abstention period. These men believed that certain behaviours, like condomless anal sex, should exclude GBM from donating. One participant thought that a “one year [deferral] is good enough” arguing that it would be difficult for people to remember their sexual activity histories and thus longer abstention periods act as a safeguard (age 26, HIV-negative, Toronto). Another man also thought 3 months might be “a little bit too soon” because it takes up to 6 months for HIV symptoms to “crop up” (age 33, HIV-negative, Vancouver). He preferred a 6-month abstention-based policy. One participant described syphilis testing results to say that he thought a 6 or 9-month deferral would be more ideal (age 34, HIV-negative, Toronto). Finally, another man questioned whether or not 3-months might be too soon: “It’s possible that I will not know what happened, given a sexual relation 3 months ago, what are the impacts on my life right now, I do not know. 3 months, I may not have time to be tested, I did not have a reason to be tested. 6 months, I think it begins to be a little more, I would not say reasonable, but you have a chance of having a confirmation if I have a doubt” (age 58, HIV-negative, Montreal).

## Discussion

In this qualitative study, we analyzed the perspectives of a diverse sample of GBM on blood donation policy. Most participants considered any MSM-specific deferral to be discriminatory and illogical [33]. Many expressed concerns with the 12-month donation policy and expressed that any policy change that maintains a risk logic that denies all MSM the ability to donate would not be addressing fundamental differences between HIV risk at the population level versus the individual level. In short, men articulated the view that MSM as a population group may have higher rates of HIV, but individuals within that population can be at significantly less risk of HIV or STIs compared to people currently eligible to donate blood [37]. Our analysis demonstrates that many GBM are highly aware and reflexive of their sexual risk levels and thus capable of self-reporting for the purposes of donation. This trend is supported by quantitative literature that has demonstrated an association between self-reporting risk behaviours with actual risk to the blood supply [38].

Our work is in line with the arguments posed by Kesby et al. [39] who suggest that when examining blood donation policy: “The dominant epidemiological paradigm of risk evaluation needs to be unpacked because it fails to address adequately the degree of fit between its epistemology of group-based deferment—grounded on aggregate epidemiological data at the population level—and the ontology of actual risk—embedded in the heterogeneous complexity of individual practice” (p23). Put differently, the general epidemiological consensus that MSM in Canada are at a higher risk for HIV and other STBBIs in comparison to most other population groups is clear. However, such a consensus does not account for the diversity of sexual practices found among MSM. There is a need for critical reflection on how a general epidemiological message about HIV risk among MSM—no doubt, an important message necessary to advance research and advocate for services to improve MSM sexual health outcomes—can be used to exclude all MSM from civic engagement, and, intentionally or not, moralize and stigmatize gay men, their sexual practices and relationships, and HIV even further. The majority of participants believe thier “actual risk” of HIV and/or other STBBIs as individuals, and not an aggregate understanding of risks for MSM populations, should be a key consideration in blood donor policy which requires screening for sexual behavior for everyone and not specific deferrals for men who have sex with men.

Participants were aware of the previous indefinite deferral for men who have sex with men. However, most did not connect this policy legacy to the tainted blood tragedy of the 1980s. This is perhaps because our sample skewed younger and most of the participants would have been children or not born during the height of this scandal. While Orsini et al. [14] are well placed to discuss the importance of remembering this significant example of government wrongdoing, many of our participants did not appear to think about issues of blood donation in relation to this legacy of institutional failure. Instead, these participants framed the initial blood ban as a response related to the AIDS crisis more broadly, and an issue of institutional homophobia and AIDS stigma in an era of insufficient knowledge and inadequate screening technology. For most of our participants, all deferral policies for MSM maintained this discriminatory character albeit in modified policy form.

Despite the varied policy responses reviewed, participants overwhelmingly interpreted any MSM-specific deferrals to be a policy problem in need of a resolution. Participants discussed two key ways they thought that current inequitable policies could change to reflect scientific knowledge on HIV and no longer be discriminatory. The first was through universal or gender-blind screening focusing on risk practices for all blood donors regardless of their sexual orientation. These practices are consistent with recent policies that have been introduced in some countries, including Italy [28]. Critiques of gender-blind screening have argued that such an approach may over-estimate risk in heterosexual donors (causing some unnecessary exclusions) and potentially under-estimate risks in some groups of MSM [37].

Indeed, though highly interested in a blood donation policy that would be applied the same to everyone regardless of sex and sexual orientation, many of our participants were also aware that the sex of the potential donor could be a significant factor in determining their risk levels. Our data does not allow us to determine the feasibility of a gender-blind approach and its ultimate effects on the donor pool. However, the reflections of our participants are important for understanding how such policies are understood and thus accepted. These debates over the practicality of gender-blind screening raise critical questions of who is prioritized in policy decision-making. Under existing policies, *all* MSM whose risk levels should make them eligible to donate are currently rejected. Under a gender-blind system, we would reject *some* heterosexual people who have safe blood to donate. Neither system is perfect, but for our participants, a gender blind policy was considered less discriminatory.

The second change participants wanted to see to blood donation policy and procedures was for deferral practices closely guided by HIV/STI testing technologies. The perceptions of our participants reveal that CBS would be well positioned to more clearly explain the practice of blood screening including why they cannot simply use more sensitive HIV tests and screen all the blood.

Participants articulated that MSM blood donor policies were an institutional outcome of homophobia and discrimination and had little to no current scientific rationale that many could understand. We concur with the work of Haire et al. who argue that a “moral imperative” [9] exists not only to maintain the safety of the blood supply but also “to ensure that differential treatment of population groups with regard to donation policy is scientifically justified” [9]. The men we spoke with argued that more equitable policy would be one more aligned with sound scientific evidence. However, participants tended to speak somewhat more favourably of a 3-month deferral policy, in part because this timeframe matched the typical window period from infection to seroconversion that has become normalized in HIV testing practices [40, 41]. While moves towards a 3-month deferral were viewed as positive by some participants—apparent stepping stones on a road to equity—the majority still considered any policy that maintained a MSM-specific deferral as representing a form of “othering” and being discriminatory. Many also saw a possible reduction to 3-months of celibacy as unrealistic and offensive.

An important limitation of our research is our specific focus on the exclusion of men based on same-sex sexual practice over other possible exclusion criteria. While this focus was helpful to complete the initial objectives of our study, we do believe that it is important to consider other intersectional grounds of GBM exclusion for donating blood—recognizing the heterogeneity of this group and that some GBM may continue to find blood donation policies inequitable because they are excluded for other reasons, such as those related to country of birth.^3^ Further analysis is also necessary to robustly account for how sex-based deferral policies may be negatively perceived by trans and non-binary people as well as the multiple reasons why some GBM may continue to find blood donation policies highly problematic in and beyond the GBM-specific deferral.

## Conclusion

Most participants believed that any MSM-specific blood deferral policy was inequitable. However, some men saw value in a 3-month deferral as an incremental step toward progress while others were critical but less resistant to the idea of a 3-month deferral. Our evidence strongly suggests that reactions to the new 3-month policy (which was recently approved by Health Canada) will be mixed and that members of GBM communities will continue to consider CBS and Héma-Québec to be discriminatory organizations, though in actuality this policy is held by Health Canada and operationalized by CBS and Héma-Québec.

Our future analyses will focus more extensively on our participants’ willingness to donate blood or plasma under modified policies as well as the specific mechanisms by which CBS and Héma-Québec can work toward regaining confidence among GBM communities. However, what is clear from the findings presented here is that GBM are looking for intelligible scientific rationales for why the policy must be different for GBM versus heterosexuals, even in the context of the newly implemented 3-month change. While some of our participants appear to be willing to accept the 3-month deferral as a step in the right direction, they want clear communication as to the rationales behind MSM-specific policies. The heterogeneity of risk among GBM led many participants to prefer individual-based versus group-based deferment policies. For these men, the higher epidemiological burden of HIV among MSM is not a convincing rationale against the backdrop of significant diversity in sexual practices and the likelihood of transmission among heterosexual men and women who can also be at-risk for HIV and other STBBIs.

## Additional file

Additional file 1: Canadian Blood Services (CBS) Qualitative Interview Guide. (DOCX 35 kb)

## Data Availability

The full qualitative transcripts for this study are not publicly available for reasons of research ethics and participant confidentiality.

## Abbreviations

GBM: Gay, bisexual, queer, and other men who have sex with men
HIRI-MSM: HIV Incidence Risk Index for Men who have Sex with Men
MSM: Men who have sex with men
PrEP: Pre-exposure prophylaxis
STBBIs: Sexually transmitted and blood borne infections

## Footnotes

1 For accuracy, we refer to the target population of the current deferral policy as being for men who have sex with men (MSM), but reference the participants we interviewed as GBM to signify the diverse ways in which they identified themselves. MSM is an epidemiological category with policy relevance, but everyday people are more likely to refer to themselves and communities through common monikers or identities like gay, bisexual, and queer [42].

2 We use the term “gender-blind” screening here to align with the language commonly used in blood donation policy debates in Canada. However, we want to acknowledge that it is somewhat inaccurate to refer to such a policy as being based on *gender*, when really the emphasis here in on the *sex* of potential donors (biological males having sex with biological males).

3 For example, when describing “Geographic Deferrals” CBS explains that “[d]onor screening procedures are exclusionary, but the exclusion is based on risk factors. It has nothing to do with race or ethnicity” [1]. We believe further intersectional research and collaboration with community groups and other researchers in this field is necessary to help elucidate the multiple experiences of perceived discrimination that may persist despite further MSM-specific policy reform efforts.
