# Supplementary material for: Gay and bisexual men’s views on reforming blood donation policy in Canada: a qualitative study
Source: BMC Public Health. 2019 Jun 17;19:772. doi: 10.1186/s12889-019-7123-4 (PMC6580549; doi:10.1186/s12889-019-7123-4)
Supplement: Supplementary file 1 — Canadian Blood Services (CBS) Qualitative Interview Guide. (DOCX 35 kb) [file 12889_2019_7123_MOESM1_ESM.docx]

**Canadian Blood Services (CBS) Qualitative Interview Guide**

**[Informed consent process completed]**

**Introductions, Socio-Demographics, and Rapport Building**

1. Would you mind starting by telling me a bit about yourself?
2. What is your age?
3. How do you usually describe your cultural background?
4. How do you usually describe your sexual identity and gender (e.g., gay, bi, queer, man, gender queer, trans, etc.)?
5. What motivated you to come in to do this interview today?
6. What is your HIV status at the time of this interview?
   1. For negative men or men with an unknown status: When was the last time you were tested for HIV?
7. Is there anything else about yourself that you think might be helpful for me to know before we move on? For example, are you currently working or studying somewhere?

**Policy Comprehension and General Opinions on Blood Donation**

1. Could you tell me what you currently know about blood donation and gay men/men who have sex with men? Do you feel knowledgeable about this issue?
   1. Probe: Do you know who is responsible for coordinating blood donation in [insert province where participant lives]?
   2. Could you tell me what you know about eligibility requirements to donate blood in general (for example, not just for gay men and other men who have with men)? [After their response, inform participant that people who have used intravenous drugs, who have Hepatitis, or who have travelled and lived in selected countries also are not able to donate blood. People under certain height/weight requirements or who have recently been ill also cannot currently donate blood.] Do you have any reflections on this?
2. Persons with a positive HIV status are not eligible to give blood. In Canada, until 2013, HIV negative/status unknown men who ever had sexual contact (oral and anal sex) with other men could not donate blood.
   1. Were you aware of this older policy? Do you know why this was the case? Do you have any opinions about this older policy?
   2. Were you aware that, for men with a negative or unknown HIV status, the policy changed to a 5-year deferral from sexual contact in 2013? [By deferral, we mean that men who had oral and/or anal sex with other men in the past 5 years were denied from being able to donate blood.]
   3. Were you aware that this policy changed to a 1-year deferral from sexual contact in 2016?
   4. Do you have any opinions about the current policy of a 1-year deferral for sexually active men who have sex with men with a negative or unknown HIV status? Do you think that this current policy is fair? Do you think that this current 1-year deferral policy is necessary to keep the blood supply safe?
3. Do you consider yourself eligible to donate blood under the current policy of a 1-year deferral? Why or why not?
4. How important is being able to donate blood to you? Probe: Was it ever important? Probe: How much would you say you desire to donate blood in the future?
5. Do you think blood donation is an important issue for the gay community? Why or why not?

**Past Experiences with Blood Donation**

1. Have you ever donated or attempted to donate blood in the past?
   1. If yes: What was that experience like for you? How many times did you donate blood? Why did you stop? Were you ever actively deferred (i.e. were you ever denied being able to give blood or had your name removed from the blood donation registry)? Were you already sexually active with men when you donated blood? If so, how did you make your decision to donate?
   2. If no: Was it because you thought you were ineligible or were there other reasons?

**Potential Policy Shifts**

1. Based on your current knowledge: who do you think should be eligible to donate blood? Who do you think should be ineligible?
   1. Probe: Do you think all gay men or men who are sexually active with other men should be able to donate blood?
   2. Probe: Do you think that HIV-positive men should be able to donate blood?

*We are now going to present you with a few potential alternatives to the current 1- year deferral policy to find out your opinions:*

1. What are your thoughts on a 6-month abstention from all sexual activity (oral and anal) for HIV negative/ status unknown men who have sex with men before being able to donate blood?
   1. *For HIV-negative participants:* Would you be willing to donate under such a policy? Do you think you would ever be eligible?
2. What are your thoughts on a 3-month abstention from all sexual activity (oral and anal) for HIV negative/ status unknown men who have sex with men before being able to donate blood?
   1. *For HIV-negative participants:* Would you be willing to donate under such a policy? Do you think you would ever be eligible?
3. How do you think other HIV prevention measures and sexual practices should factor into eligibility requirements for blood donation for men who have sex with men?
   1. For example, should monogamy be a factor? HIV risk reduction measures such as the use of pre-exposure prophylaxis (PrEP)? The use of condoms? Viral load and undetectability if having sex with someone who is HIV positive? The kinds of sex someone is having (oral vs. anal; topping vs. bottoming)?
4. Instead of a timed deferral policy (e.g. needing to have a 3 or 6 month abstention from sex) for men who have sex with men, do you think blood donation policy should be the same for heterosexually and homosexually active (gay and straight) men and women, based on their specific risk behaviours?
5. Let us take a moment to recap: of all these different policy alternatives and ideas that we have just discussed, in your opinion, what is the most acceptable (both fair and safe for the blood supply) policy for blood donation and men who have sex with men?
   1. Probe: Put differently: how would you like the policy to look going forward? What do you not want to see the policy look like going forward?
6. *For HIV-negative participants:* Are you familiar with the idea of plasma donation?
   1. Probe: Here is a quick background. Plasma refers to the liquid portion of your blood. So it is blood without red blood cells, white blood cells, and platelets. Plasma is collected through a centrifuge machine that removes cells from the plasma and then returns these cells back to your body. The process takes about an hour and a half to complete. A person registering in a plasma donor program is expected to donate plasma regularly. How much would you say you desire to donate plasma in the future? How important is being able to donate plasma to you?
   2. Because plasma donations can be stored for much longer than regular blood donations, it is possible to do additional testing on plasma at a later date to confirm the donation’s safety before it is supplied to those in need. Because of these extra safety mechanisms, one potential blood donation policy being explored is allowing HIV negative/status unknown gay men and other men who have sex with men to donate plasma even if they have had sex more recently and would not be able to donate blood. This process would involve having to donate plasma multiple times for eligibility. What are your thoughts on the ability to donate plasma even if you can’t donate whole blood? Under this policy shift, would you be interested in donating plasma?

*For HIV-positive participants:* Are you familiar with the idea of plasma donation?

- 1. Probe: Here is a quick background. Plasma refers to the liquid portion of your blood. So it is blood without red blood cells, white blood cells, and platelets. Plasma is collected through a centrifuge machine that removes cells from the plasma and then returns these cells back to your body. The process takes about an hour and a half to complete. A person registering in a plasma donor program is expected to give plasma donation regularly. How important do you think being able to donate plasma is for gay men?
  2. Because plasma donations can be stored for much longer than regular blood donations, it is possible to do additional testing on plasma at a later date to confirm the donation’s safety before it is supplied to those in need. Because of these extra safety mechanisms, one potential blood donation policy being explored is allowing HIV negative/status unknown gay men and other men who have sex with men to donate plasma even if they have had sex more recently and would not be able to donate blood. This process would involve having to donate plasma multiple times for eligibility. What are your thoughts on the ability to donate plasma even if someone can’t donate whole blood? Under this policy shift, do you think gay men would be interested in donating plasma?

**Personal Risk Evaluation**

1. *For HIV-negative participants*: Could you describe to me how “at-risk” you believe you currently are for contracting HIV? For other sexually transmitted infections? Why do you believe this is the case?
2. *For HIV-negative participants*: Could you describe to me, in general, what your current sexual practices have been in the last year?
   1. What forms of HIV prevention do you usually use?
   2. Do you have sex with HIV-positive guys? Do you have sex with guys whose HIV-statuses you don’t know?
   3. How often would you say you have concerns about your HIV status? How frequently do you usually get tested?
3. *For HIV-negative participants*: Do you feel that your current “risk levels” should make you eligible for donating blood? Why or why not?
4. *For HIV-negative participants*: Do you think that your current “risk levels” might change in the future? How do you think that might impact your eligibility and willingness to donate blood?

**Screening and Deferral Practices**

1. Do you have suggestions about how Canadian Blood Services can screen potential donors without alienating them? Are there any words, phrases or questions that you think should be avoided—that is, what are some things that you might find offensive or less offensive?
2. How do you feel about the following screening questions being used:
   1. “In the last 12 months, have you had sex with another man?”
   2. “Have you had sex with anyone who has AIDS or has tested positive for HIV or AIDS?”
3. We want to get a sense of whether or not you find the following screening questions acceptable/appropriate and how you would interpret them if asked. Would you be willing and able to answer the following questions if being screened for blood donation? :
   1. “How many men have you had sex with in the last 6 months?
      1. How do you interpret this question? Would you be comfortable answering this question? Would you find being asked this question offensive?
   2. “Have you had anal sex without a condom in the last six months? How about as a receptive anal sex partner?”
      1. How do you interpret these questions? Would you be comfortable answering these questions? Would you find being asked these question offensive?
   3. “How many of your male sex partners were HIV positive?”
      1. How do you interpret this question? Would you be comfortable answering this question? Would you find being asked this question offensive?
   4. “Have you been using pre-exposure prophylaxis medications (PrEP)?”
      1. How do you interpret this question? Would you be comfortable answering this question? Would you find being asked this question offensive?
   5. “Have you had a sexually transmitted infection (STI) in the last six months?”
      1. How do you interpret this question? Would you be comfortable answering this question? Would you find being asked this question offensive?
4. Do you think gay men are going to be willing to donate blood under modified policies?
   1. What do you think Canadian Blood Services can do to authentically improve its relationship with the gay community?
5. Do you have any other comments, questions or feedback?

**Thank you for your participation in this interview.**
